# Prediction of frailty in community older adults based on machine learning: a systematic review and meta-analysis

**DOI:** 10.3389/fpubh.2025.1667792

**Published:** 2026-01-12

**Authors:** Yifan Ou, Dandan Jiang, Pan Li, Wen Zhang, Yutong Zhou, Yao Chen, Xinhong Yin

**Affiliations:** 1 School of Nursing, University of South China, Hengyang, Hunan, China; 2 The Second Hospital, University of South China, Hengyang, Hunan, China

**Keywords:** aged, artificial intelligence, community, frailty, machine learning, prediction model, systematic review/meta-analysis

## Abstract

**Background:**

An increasing number of predictive models for frailty in community-dwelling older adults are now being developed using machine learning methods. Differences between model performances limit their practical application. Therefore, we conducted a systematic review and meta-analysis to summarize and evaluate the performance and clinical applicability of these risk prediction models.

**Methods:**

PubMed, Web of Science, Embase, Cochrane Library, Scopus, CINAHL, SinoMed, VIP, CNKI, and Wanfang were searched. The search time was from the database establishment to June 10, 2025. The PROBAST+AI assessment tool was used to assess the study quality, and a meta-analysis of the area under the curve (AUC) was performed using Stata18.0 software.

**Results:**

A total of 10 studies were included, and 45 Machine Learning (ML) models were developed, of which 36 models were developed for internal validation and 9 for external validation. In the internal validation set, the pooled AUC for baseline frailty prediction studies was 0.878 (95% CI 0.799, 0.958), while the pooled AUC for longitudinal frailty prediction studies was 0.730 (0.670, 0.790). When all studies were pooled without distinguishing prediction time points, the overall pooled AUC was 0.786 (95% CI 0.697, 0.875).

**Conclusion:**

Although most of the included models had good discrimination and calibration, the overall quality and applicability of the current study are still problematic. In future studies, researchers should follow the TRIPOD+AI statement and the PROBAST+AI list to construct high-quality, more applicable predictive models.

**Systematic review registration:**

https://www.crd.york.ac.uk/PROSPERO/view/CRD420251071061, identifier CRD420251071061.

## 1 Introduction

As people around the world are living longer, populations are aging much faster than ever before. According to United Nations data, by 2050, the number of people over the age of 60 will increase to 2.1 billion worldwide (1). Frailty is primarily characterized by a decrease in physiological reserves and resistance to stressors as we age (2). The global prevalence of frailty among older people in the community is 43.3% (3). Not only does frailty affect the physical health and quality of life of older people, but it can also pose significant challenges to clinical practice and public health (4). In addition to this, frailty increases the incidence of adverse health outcomes such as falls (5), hospitalization (6), and death (7) among community-dwelling older adults.

Relevant research suggests that exercise interventions, nutritional support (8), and the development of management plans through comprehensive geriatric assessments (9) can prevent, delay, or reverse frailty in community-dwelling older adults (10). Due to the complex pathophysiological mechanisms and diverse manifestations of frailty, it often presents with atypical symptoms clinically, making it prone to being overlooked and underdiagnosed in routine medical care (11). Furthermore, frailty typically progresses insidiously, and by the time a definitive diagnosis is made in the late stages, the optimal window for intervention is often missed. Currently, commonly used frailty assessment tools, such as the Fried Frailty Phenotype and Frailty Index, are primarily employed to screen individuals already in a frail state, rather than identifying those at high risk of frailty (12). The use of validated measurement tools to identify frailty and, thus, early management of frailty is considered a critical step in preventing frailty (13, 14). Predictive models can estimate an individual’s risk of a specific clinical outcome or event within a specific time frame based on multiple predictors (15), which can help to screen at-risk populations so that early interventions can be made to prevent disease progression (16).

In recent years, research on developing, validating, and updating predictive models in this field has increased to enable early identification of frailty-prone older adults individuals within communities (17). These models assess the likelihood and risk of frailty onset among community-dwelling older adults based on multidimensional predictors including demographic characteristics, laboratory indicators, and clinical and psychological assessments. Relevant studies indicate that machine learning not only enhances predictive accuracy by leveraging big data or multidimensional datasets but also facilitates early disease detection and promotes targeted preventive interventions (18, 19). Furthermore, machine learning efficiently processes large-scale datasets, generates hypotheses from data, and identifies underlying patterns. Advances in machine learning methodologies have made developing predictive models using these techniques faster and more accessible (20). In summary, machine learning algorithms hold potential value in identifying frailty risk among community-dwelling older adults. However, due to the diversity of ML algorithms, variations in sample sizes and datasets, differences exist in the predictive performance of different models. The risk of bias in predictive models and their applicability in clinical practice requires further evaluation.

With the widespread application of machine learning in the health sector, research on frailty prediction models for older adults has been increasing. Existing systematic reviews have primarily focused on traditional statistical models such as logistic regression and Cox proportional hazards models (17). While one study most closely aligned with this topic systematically reviewed machine learning-based frailty prediction models for community-dwelling older adults, revealing widespread deficiencies in reporting standards and methodological quality within the field, it did not conduct correlation analyses on model performance (21). Although researchers are employing reporting guidelines and quality assessment tools to address modeling deficiencies and reduce bias, model predictive performance remains inconsistent, casting uncertainty over their clinical applicability. Therefore, in this study, the latest risk prediction model evaluation tool, PROBAST+AI (22), was used to evaluate frailty prediction models constructed for community-dwelling older adults using machine-learning methods, and a meta-analysis was performed to analyze the AUC of the prediction models. It is expected to provide valuable guidance and reference for clinical practitioners in the development of frailty risk prediction models for community-dwelling older adults.

## 2 Methods

This department’s research adheres to the Transparent Reporting of a Multivariable Prediction Model for Individual Prognosis or Diagnosis-Systematic Reviews and Meta-Analysis (TRIPOD-SRMA) (23) and the Preferred Reporting Items for Systematic Reviews and Meta-Analyses (PRISMA) 2020 guidelines (24). This study has been registered with PROSPERO (CRD420251071061).

### 2.1 Search strategy

We conducted a comprehensive search of multiple databases, including PubMed, Web of Science, Embase, Cochrane Library, Scopus, SinoMed, China National Knowledge Infrastructure (CNKI), Wanfang Database, China Science and Technology Journal Database (VIP), Cumulative Index to Nursing and Allied Health Literature (CINAHL). The search was conducted from the time of database construction to June 10, 2025. The objective was to identify studies that used machine learning methods to predict the onset of frailty in community-dwelling older adults. The search strategy used a combination of keywords and subject terms such as “machine learning,” “artificial intelligence,” “predictive models,” “risk prediction modelling,” “frailty,” “aged”, “community”, etc. The detailed search strategy is shown in Supplementary Table 1, and the reference lists of the included literature were carefully reviewed to ensure comprehensiveness.

For systematic evaluation, we used the PICOTS system recommended by the CHARMS (Critical Appraisal and Data Extraction for Systematic Reviews of Prediction Modeling Studies) list (25). The system helps frame the purpose of the review, the search strategy, and the inclusion and exclusion criteria for the studies. The key items of PICOTS are described as Table 1.

**Table 1:** Key projects of PICOTS.

PICOTS	Description
P (population)	Community-dwelling older adults aged ≥60 years
I (Index prediction model)	Predictive models developed and/or validated using machine learning algorithms
C (Comparative)	A comparative study of machine learning algorithms versus other machine learning algorithms, traditional statistical analysis, and clinical scoring tools
O (Outcomes)	The predicted outcome is frailty
T (Timing)	Studies on cross-sectional and/or longitudinal prediction
S (Setting)	Research conducted in community settings

### 2.2 Inclusion and exclusion criteria

Based on the aforementioned research framework, we established the following inclusion and exclusion criteria:

Inclusion criteria: (1) Population: Community-dwelling older adults aged ≥60 years; (2) Predictive Model Type: Studies must develop or validate at least one machine learning-based predictive model, or compare machine learning algorithms with other machine learning algorithms, traditional statistical analyses, or clinical scoring tools, and must report at least one model performance metric (discrimination and/or calibration); (3) Study design: cohort studies, cross-sectional studies, and case–control studies; (4) Study outcome: predicted outcome of frailty (comprehensive physical frailty), with frailty defined based on recognized standards or tools.

Exclusion criteria: (1) Studies not reporting the predictive model development process; (2) Studies limited to cognitive frailty or social frailty; (3) Literature not in English or Chinese; (4) Studies where full text was unavailable; (5) Case reports, reviews, conference abstracts, or guidelines; (6) Studies employing only traditional statistical models without machine learning algorithms, or studies conducted in non-community settings such as hospitals, clinics, or long-term care facilities; (7) Studies failing to report model performance metrics.

### 2.3 Screening of articles

First, all literature retrieved from various databases was imported into EndNote X9 software for management, with duplicate records automatically removed. Subsequently, two independent researchers conducted an initial screening of the remaining literature’s titles and abstracts to exclude obviously irrelevant studies. Finally, the same two researchers reviewed the full texts of the initially screened articles against predefined inclusion and exclusion criteria to determine the final list of included studies. Any disagreements were resolved through discussion or adjudication by a third researcher. Additionally, we carefully examined the reference lists of all eligible studies to ensure the completeness of the included literature.

### 2.4 Data extraction

The search results were screened independently by two authors according to the CHARMS list. In case of disagreement, it was decided through discussion or by the third author. The information extracted from the selected articles included: (1) Basic information of the study: including information on the data source, country, year of publication, authors, sample size, outcome, outcome assessment tool, and candidate predictors. (2) Model information: including the extraction of information such as predictor screening methods, model construction methods, missing data handling methods, continuous variable handling methods, evaluation model indicators, and predictors finally included in the model.

### 2.5 Quality assessment

Quality, potential risk of bias, and study applicability of the included literature were assessed using the PROBAST+AI evaluation tool (22), a newly developed assessment tool for evaluating the quality, risk of bias, and applicability of regression- or artificial intelligence-based predictive models. It includes four domains: participants and data sources, predictors, outcomes, and analyses; a total of 34 signaling questions (16 for model development and 18 for model evaluation) and 6 applicability items (3 each for model development and model evaluation) were addressed. Signaling questions are answered as yes, probably yes, no, probably no, no information, or, when appropriate, not applicable. Quality concerns (for model development) are judged as low, high, or unclear, and risk of bias (for model evaluation) is judged as low, high, or unclear.

### 2.6 Statistical analysis

First, we summarized the AUC values and their 95% confidence intervals (95% CI) for each study. Due to the inconsistent time windows for frailty prediction across the included studies—three studies assessed baseline prediction, six studies assessed longitudinal prediction, and one study assessed both baseline and longitudinal prediction—we conducted meta-analyses based on the prediction window (baseline vs. longitudinal) and stratified analyses based on the model construction method. For studies that did not report the standard error (SE) or 95% CI of AUC, the Hanley and McNeil formula was used to estimate the SE and 95% CI (26, 27). Considering that the included studies may have heterogeneity among study designs, ML models, and predictors. Therefore, the AUC of each study was combined in the meta-analysis using the Der Simonian and Laird random effects model (28). Heterogeneity between studies was assessed using the Cochrane *Q*-test and the *I*^2^ statistic (*I*^2^ ≥ 50% or *p* ≤ 0.10) to determine whether to use a fixed-effects model or a random-effects model (29). All statistical analyses were performed using Stata18.0 software and results were considered statistically significant at *p* < 0.05.

## 3 Results

### 3.1 Search results

**Figure 1:**
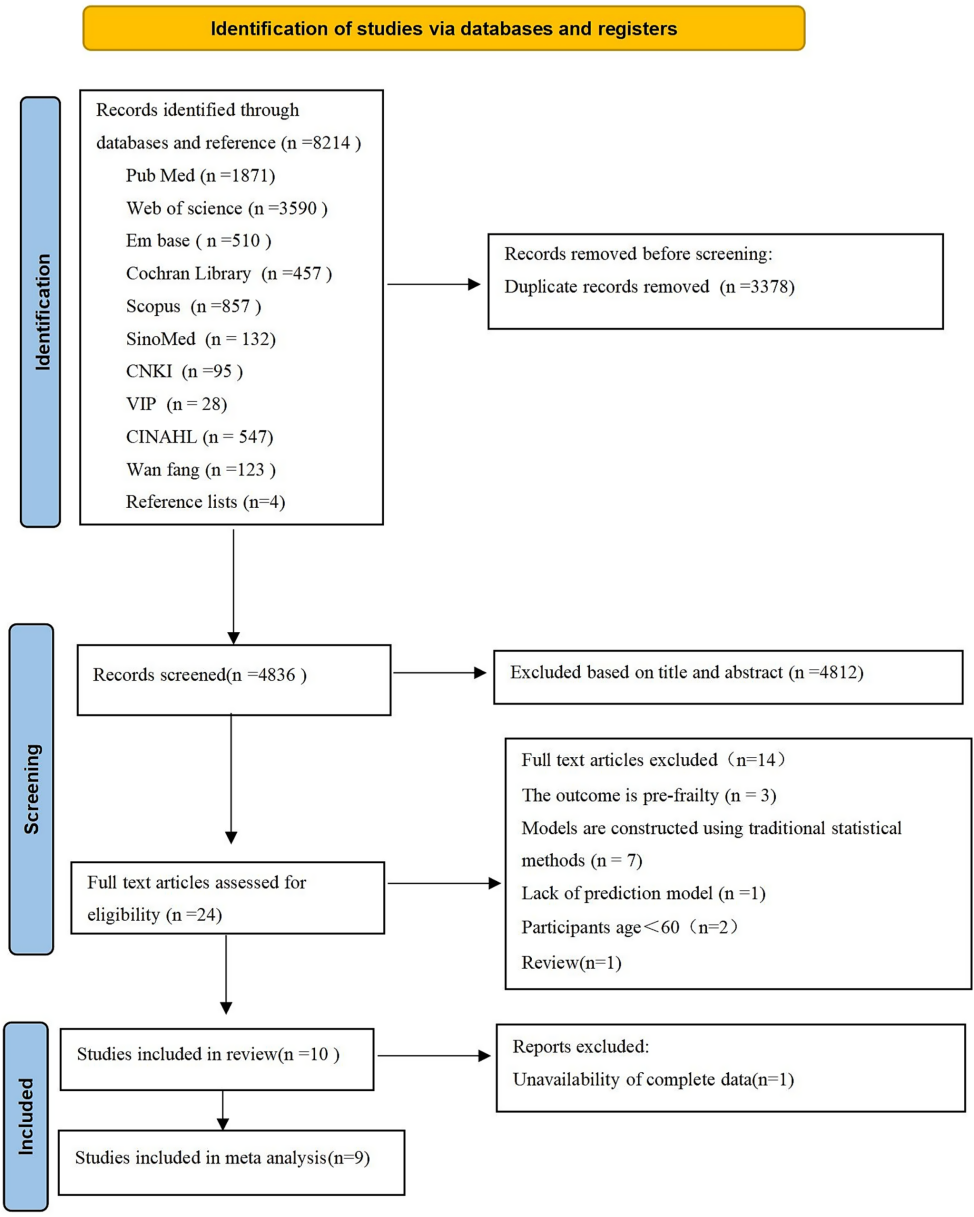
Shows the Preferred Reporting Items for Systematic reviews and Meta-Analyses (PRISMA) 2020 flowchart depicting the comprehensive search process and results.

Through systematic searching, we retrieved 8,210 articles from 10 databases. We also identified 4 studies by reviewing reference lists. After deduplication using EndNote X9 literature management software, 4,836 articles remained. Initial screening based on titles and abstracts led to the exclusion of 4,812 articles, leaving 24 articles for full-text eligibility assessment. After reviewing the full texts, we excluded 14 articles (reasons for exclusion detailed in Figure 1). Ultimately, 10 articles were included in this study (30–39). Figure 1 shows the Preferred Reporting Items for Systematic reviews and Meta-Analyses (PRISMA) 2020 flowchart depicting the comprehensive search process and results.

### 3.2 Basic characteristics of the included literature

Ten studies were included in this study with publication dates of 2022–2025, all of which were in English. Most studies were cohort-based, including six prospective cohort studies, one retrospective cohort study, one cross-sectional cohort study, and two cross-sectional studies. Data for seven studies originated from single-center databases, while the remaining three studies utilized data collected by researchers from community sources. Among studies developing models using databases, data were sourced from China (*n* = 5), Japan (*n* = 1), and South Korea (*n* = 1). Study sample sizes ranged from 214 to 14,540 participants, with 28–4,883 outcome events recorded. Prevalence rates varied between 5.33% and 64.77%.

Candidate predictors numbered between 16 and 56, most commonly comprising demographic characteristics, clinical laboratory data, and lifestyle factors. Regarding frailty assessment, the most frequently used tools were the Fried Phenotype (*n* = 4) and Frailty Index (*n* = 3), with others including Kihon (*n* = 1), Study of Osteoporotic Fractures (SOF) Index (*n* = 1), and Tilburg Frailty Indicator (TFI) (*n* = 1). Among these, five studies employed a binary classification of “frail” and “non-frail” to define outcomes, while the remaining five studies included a “pre-frail” state in their outcome definitions. The detailed characteristics of the included models are shown in Table 2.

**Table 2:** Overview of basic data of the included studies.

Author (year)	Country	Study design	Participants	Data source	Time windows	Main outcome	Frailty cases/sample size (%)	Candidate predictors	Outcome measures
Mengjiao Yang 2024 (37)	Japanese	Cross-sectional and longitudinal study	Aged ≥65 years Mean age 73.7 Female 49.7%	Community Empowerment and Care for Wellbeing and Healthy Longevity: Evidence from a Cohort Study	Baseline and Longitudinal	Frailty	Cross-sectional: 95/673 (14.1%) Longitudinal: 90/373 (24.1%)	34 (Socio-demographic characteristics, Lifestyle characteristics, Disease characteristics, Physical condition, Psychological condition, Social relationships)	Kihon
Qinqin Liu 2023 (34)	China	Prospective cohort study	Aged ≥60 years Mean age 66.7 Female 47.3%	China Health and Retirement Longitudinal Study	Longitudinal	Frailty	Derivation cohort: 766/2,241 (34.2%) Internal validation cohort: 195/561 (34.8%) External validation cohort: 557/1,721 (32.4%)	37 (Sociodemographic, Clinical, lifestyle-related and Cognitive psychological factors)	Fried frailty Phenotype
Shaoyi Fan 2023 (31)	China	Prospective cohort study	Aged 60–95 years Mean age 68.9 Female 72.9%	Four community centers in Guangzhou	Baseline	Frailty	28/214 (13.1%)	16 (GCA, Walking test)	Fried frailty Phenotype
Yafei Wu 2022 (36)	China	Retrospective cohort	Aged ≥65 years Mean age 77.0 Female 52.0%	Chinese Longitudinal Healthy Longevity and Happy Family Study	Longitudinal	Frailty	713/4,083 (17.4%)	56 (Socio-demographic characteristics, Lifestyles, self-reported health and Objective examination)	Frailty Index
Heeeun Jung 2023 (33)	Korea	Cross-sectional	Aged 70–84	Korea frailty and aging cohort study	Baseline	Frailty	216/1,482 (25.0%)	32 (Socio-demographic domain, Physical domain, Biological domain, Lifestyle domain, Health condition domain, Medical domain, Psychological domain, Social domain)	Fried frailty Phenotype
Yin Yuan 2022 (38)	China	Prospective cohort study	Aged ≥ 60 years Mean age 72.5 Female 60.0%	The residents from community and nursing homes in Fujian Province	Longitudinal	Frailty	711/1,981 (35.8%)	32 (Demographic characteristic, Lifestyle factors, Physical function, Psychophysiological, Social support, Social environment, Physical examination, Laboratory indicators)	Fried frailty Phenotype
Yongfei Dong 2025 (30)	China	Prospective cohort	Aged ≥65 years	Chinese Longitudinal Healthy Longevity Survey	Longitudinal	Frailty	Training: 1,493/4,878 (30.6%) External Validation 1: 968/3,840 (25.2%) External Validation 2: 1,531/5,822 (26.3%)	22 (Socio-demographic, Socio behavioral, Medical measures factors)	Frailty Index
Lin Qi 2025 (35)	China	Cross-sectional	Aged ≥60 years Female 52.5%	Communities in eastern China	Baseline	Frailty	818/1,263 (64.7%)	17 (Demographic characteristic, Frailty indicators)	Tilburg Frailty Indicator
Wei Zhang 2024 (39)	China	Prospective cohort	Aged ≥65 years	Chinese Longitudinal Healthy Longevity Survey	Longitudinal	Frailty	912/6,997 (13.0%)	46 (Demographic data, Health-related risk factors, Risk factors for chronic diseases)	SOF index
Li Huang 2024 (32)	China	Prospective cohort	Aged ≥65 years Mean age 85.3/85.3/89.5 Female 54.1%/54.8%/49.6%	Chinese Longitudinal Healthy Longevity Survey	Baseline	Frailty	4,883/14,925 (29.3%)	54 (Demographic characteristics)	Frailty Index

### 3.3 Characteristics of the model development

Table 3 summarizes detailed information on model development characteristics. In terms of variable treatment, four studies converted continuous variables to categorical variables (32, 35, 37, 38). In terms of missing data treatment, four studies used multiple imputation (31, 34, 37, 39), two studies excluded missing data (33, 35), three studies did not report information about missing data (30, 32, 38), and one study used the miss Forest method for missing data (36). Among the methods for predictor screening, commonly used approaches include Lasso regression (37, 38), random forest method (33, 36), and univariable analysis (31, 35). The number of predictors included in the final model ranged from 5 to 16, covering a total of 61 predictors. The most common predictor was age (*n* = 8), followed by cognitive function (*n* = 5) and depression (*n* = 3).

**Table 3:** Overview of the information of the included prediction models.

Author (year)	Missing data handling	Continuous variable processing method	Variable selection	Model development method	Validation method	Calibration method	Model performance (AUC)	Final predictors	Model presentation
Mengjiao Yang 2024 (37)	Multiple imputation	Categorical variable	LASSO regression	Bayesian networks	Internal validation (tenfold cross-validation)	B: Hosmer-Lemeshow *p* = 0.395	B: Cross-section: 0.943 (0.913, 0.974) Longitudinal: 0.722 (0.656, 0.788)	7:≥75 years Having physical dysfunction Polypharmacy Dementia Musculoskeletal disorders Depression Social relationships	Graphical score char
Qinqin Liu 2023 (34)	Multiple imputations	Continuous variable	LASSO regression and Expert opinion	LR SVM RF XGBoost	Internal validation (random split) and External validation	Hosmer-Lemeshow test LR: 0.614/0.919/0.008 SVM: 0.134/0.138/0.039 RF: <0.001/0.249/0.107 XGBoost: 0.600/0.120/<0.001 Brier score: LR: 0.207/0.206/0.214 SVM: 0.209/0.207/0.217 RF: 0.193/0.205/0.213 XGBoost: 0.200/0.200/0.216	A: LR: 0.669 (0.645, 0.692) SVM: 0.665 (0.641, 0.688) RF: 0.748 (0.727, 0.769) XGBoost: 0.700 (0.628, 0.723) B: LR: 0.676 (0.628, 0.723) SVM: 0.676 (0.629, 0.722) RF: 0.687 (0.641, 0.733) XGBoost: 0.701 (0.655, 0.746) C: LR: 0.614 (0.586, 0.642) SVM: 0.611 (0.583, 0.640) RF: 0.621 (0.584, 0.640) XGBoost: 0.612 (0.584, 0.640)	14: Waist circumference Age Cognitive function SRH Material health Medical insurance Current residence location Pension insurance Housing tenure Afternoon napping, Eating habit Depression Hearing impairment Lung diseases	Online clinical support, Nomogram
Shaoyi Fan 2023 (31)	Multiple imputations	Continuous variable	Univariable analysis	RF DT NN SGD NB	Internal validation (random split)	–	B: RF: 0.926 DT: 0.850 NN: 0.761 SGD: 0.834 NB: 0.761	5: Large-step walking speed Average step size Age Total step walking distance MMSE	–
Yafei Wu 2022 (36)	Miss Forest	Continuous variable	Recursive feature elimination (RFE) and 10-fold cross-validation based on random forest	NB LR DT SVM ANN, RF XGB	Bootstrap internal validation	B: Brier score: LR: 0.205 NB: 0.161 DT: 0.208 SVM: 0.181 RF: 0.143 XGB: 0.134 ANN: 0.193	B: LR: 0.728 (0.687, 0.769) NB: 0.722 (0.687, 0.769) DT: 0.612 (0.580, 0.643) SVM: 0.661 (0.619, 0.703) RF: 0.702 (0.659, 0.745) XGB: 0.677 (0.659, 0.745) ANN: 0.640 (0.595, 0.685)	11: Marital status ADL; IADL; MMSE Hypertension Childhood starvation Heart disease Weight Stroke Cerebrovascular disease Medical costs payer	–
Heeeun Jung 2023 (33)	Remove	Continuous variable	Random forest model analysis	LR KNN SVM Gaussian NB RF	Internal validation (random split)	–	F1score: LR: 0.920 KNN: 0.820 SVM: 0.870 Gaussian NB: 0.880 RF: 0.910	16: Age Low education level Residence Low calf circumference TUG duration SPPB; Hs-CRP; eGFR Serum creatinine Triglyceride RBC 25-hydroxy vitamin D Number of medications Risk of malnutrition EQ-5D index Depressive symptoms	–
Yin Yuan 2022 (38)	–	Categorical Variable	LASSO regression	Bayesian networks	Internal validation (random split)	Calibration plots and Hosmer-Lemeshow (*p* = 0.160/0.341)	B: 0.701	6: Age Nutritional status IADL Balance capacity Social support	Graphical score char
Yongfei Dong 2025 (30)	–	Continuous variable	Stepwise-multivariate and Cox model	XG Boost GBM Cox Boost.	Bootstrap Internal validation and external validation	Brier score: <0.25	B: XG Boost: 0.830 (0.810, 0.840) GBM: 0.820 (0.800, 0.830) CoxBoost: 0.800 (0.780, 0.820) Cox model: 0.800 (0.780, 0.820) C: XG Boost: 0.850 (0.830, 0.870) GBM: 0.850 (0.830, 0.870) CoxBoost.: 0.850 (0.830, 0.870) Cox model: 0.850 (0.830, 0.870)	9: Age BMI Cognitive function, Gender Ethnicity Education Natural teeth status Smoking status Occupation	Nomogram
Lin Qi 2025 (35)	Remove	Categorical Variable	Univariable analysis	RF XG Boost DT	Internal validation (random split)	–	A: RF: 0.990 XGBoost: 0.810 DT: 0.780 B: RF: 0.750 XGBoost: 0.720 DT: 0.660	9: Age BMI Monthly income Living arrangement Visit frequency Pension insurance Smoking status Number of chronic diseases Type of medication	–
Wei Zhang 2024 (39)	Multiple imputation	Continuous variable	Stepwise regression	RF LR	Internal validation (random split)	–	A: RF: 0.970 LR: 0.780 B: RF: 0.770 LR: 0.760	10: Age Household status Marital status Self-reported health status MMSE Exercise ADL Sleep duration Hypertension Pulmonary disease	Nomogram
Li Huang 2024 (32)	–	Categorical Variable	Lasso regression and Boruta algorithm Random forest and classifier score	LR RF SVM XG Boost SHLNN	Internal validation (random split) and external validation	–	B: LR: 0.974 RF: 0.967 SVM: 0.974 XGBoost: 0.974 SHLNN: 0.974 C: LR (2,011): 0.963 (0.946, 0.979) LR (2,014): 0.977 (0.968, 0.987) AUC (Women): 0.971 AUC (Man): 0.975	10 key features: Able to go shopping by yourself? Able to walk one kilometer? Able to carry 5 kg weight? Able to make food by yourself? Able to crouch and stand for three times? Able to wash clothes by yourself? Able to take public transport? Able to pick up a book from the floor? Able to stand up from sitting in a chair? Visual function	Website

–, No information; A, Training set; B, Internal validation; C, External validation. RF, Random forest; LR, Logistic regression: SVM, Support vector machine; XGBoost, eXtreme Gradient Boosting; NN, Neural network; SGD, Stochastic gradient descent; NB, Naïve Bayes; ANN, Artificial neural network; KNN, K-nearest neighbors; Gaussian NB, Gaussian Naïve Bayes; GBM, Gradient Boosting Machine; SHLNN, Single-hidden-layer Neural Net; DT, Decision tree; MMSE, Mini Mental State Examination score; IADL, Instrumental activities of daily living.

### 3.4 Characteristics of model validation

Internal validation was performed in all 10 included studies, with three studies performing both internal and external validation (30, 32, 34) and two studies performing two external validations. The methods used in internal validation were random split (*n* = 7), bootstrap (*n* = 2), and 10-Fold Cross-Validation (*n* = 1). The three studies that conducted external validation used the time validation method. Specific information about modeling is shown in Table 3.

### 3.5 Performance of the models

Table 3 summarizes detailed information on model performance. A total of 45 models were constructed using 16 methods across the 10 studies. The most used methods were Random Forest (RF, *n* = 8), Logistic Regression (LR, *n* = 7), Extreme Gradient Boosting (XGB, *n* = 7), and Support vector machine (*n* = 5). In the included studies, the performance of the models was assessed mainly through differentiation and calibration methods. Nine of the studies measured model discrimination by AUC, and one study did not report model discrimination and used the F1 score to measure model performance.

Five studies reported model calibration methods. In the model construction phase, two studies assessed calibration using the Brier score and their constructed models all scored <0.25, and 2 studies assessed calibration using the Hosmer-Lemeshow (H-L) test, with the majority of the models demonstrating good calibration performance (*p* > 0.05). In internal validation, studies using the H-L test and Brier score also showed good calibration performance (*p* > 0.05, Brier score < 0.25). In external validation, two studies assessed predictive model calibration using the Brier score (*n* = 2) and H-L test (*n* = 1), respectively.

### 3.6 Form of model presentation

Six studies reported on the presentation form of the model. Of these, one study presented the model in Nomogram and Online clinical supports, and another presented it in Nomogram and Prognostic index, respectively. The models of the remaining four studies were presented as graphs (*n* = 2), websites (*n* = 1), and column line graphs (*n* = 1), respectively.

### 3.7 Interpretability of predictive models

Among the 10 included studies, two employed the SHAP method to quantify feature contributions, two conducted interpretable model inference based on Bayesian networks, four presented primary predictors through feature importance ranking, one visualized predictor weights using a column chart, and one study utilized both feature importance ranking and column charts. To further summarize interpretive findings across models, we compiled the top five key predictors reported in the included studies. Results indicated that age (*n* = 7), cognitive function (*n* = 5), and ADL (*n* = 3) were the most frequently occurring predictors. Supplementary Table 3 summarizes the interpretability analysis methods used in the study and the top five key predictors identified by each method.

### 3.8 Evaluation of the quality of the literature

**Figure 2:**
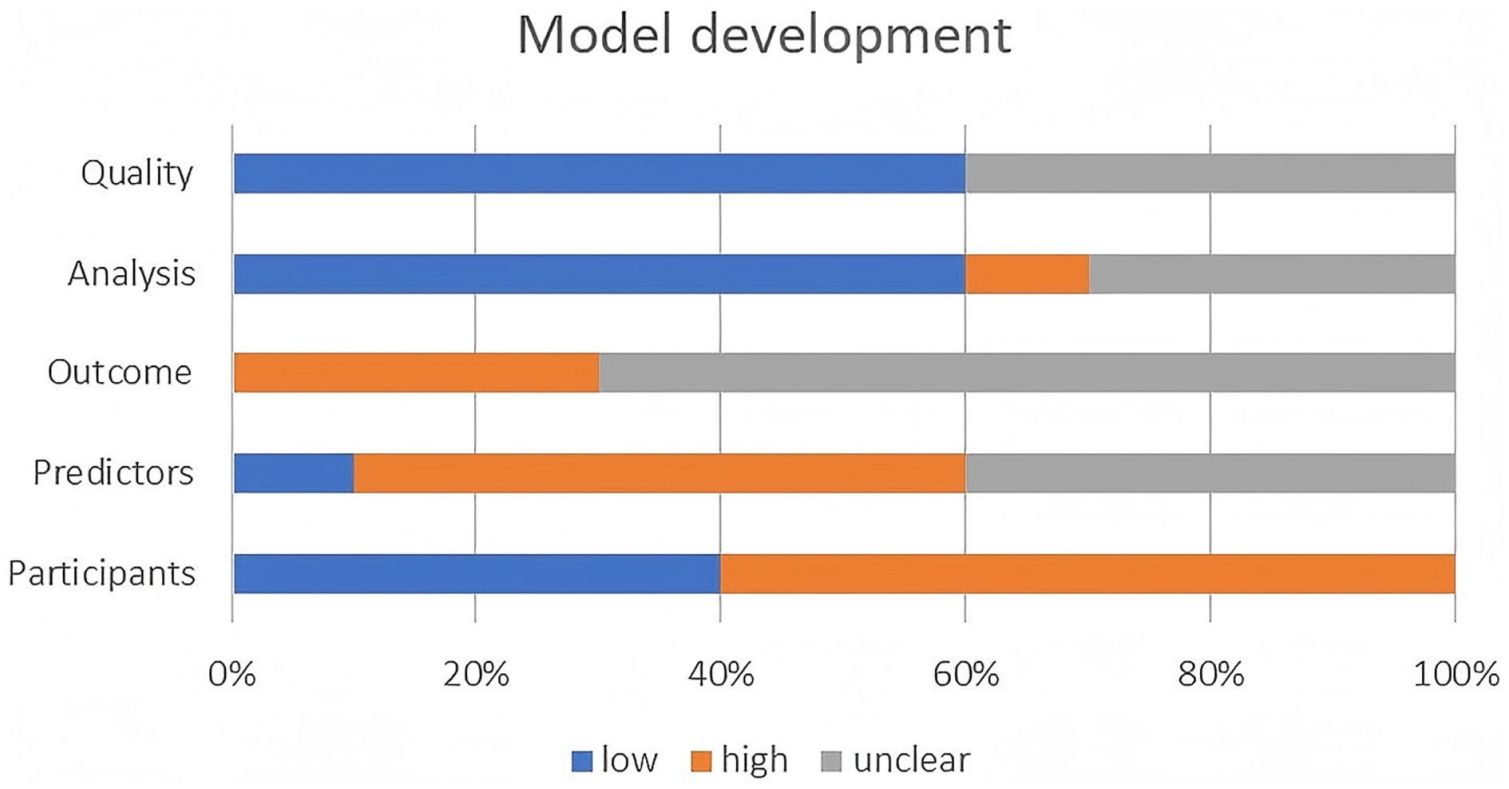
Bar chart of PROBAST+AI quality assessment results included in the study.

**Figure 3:**
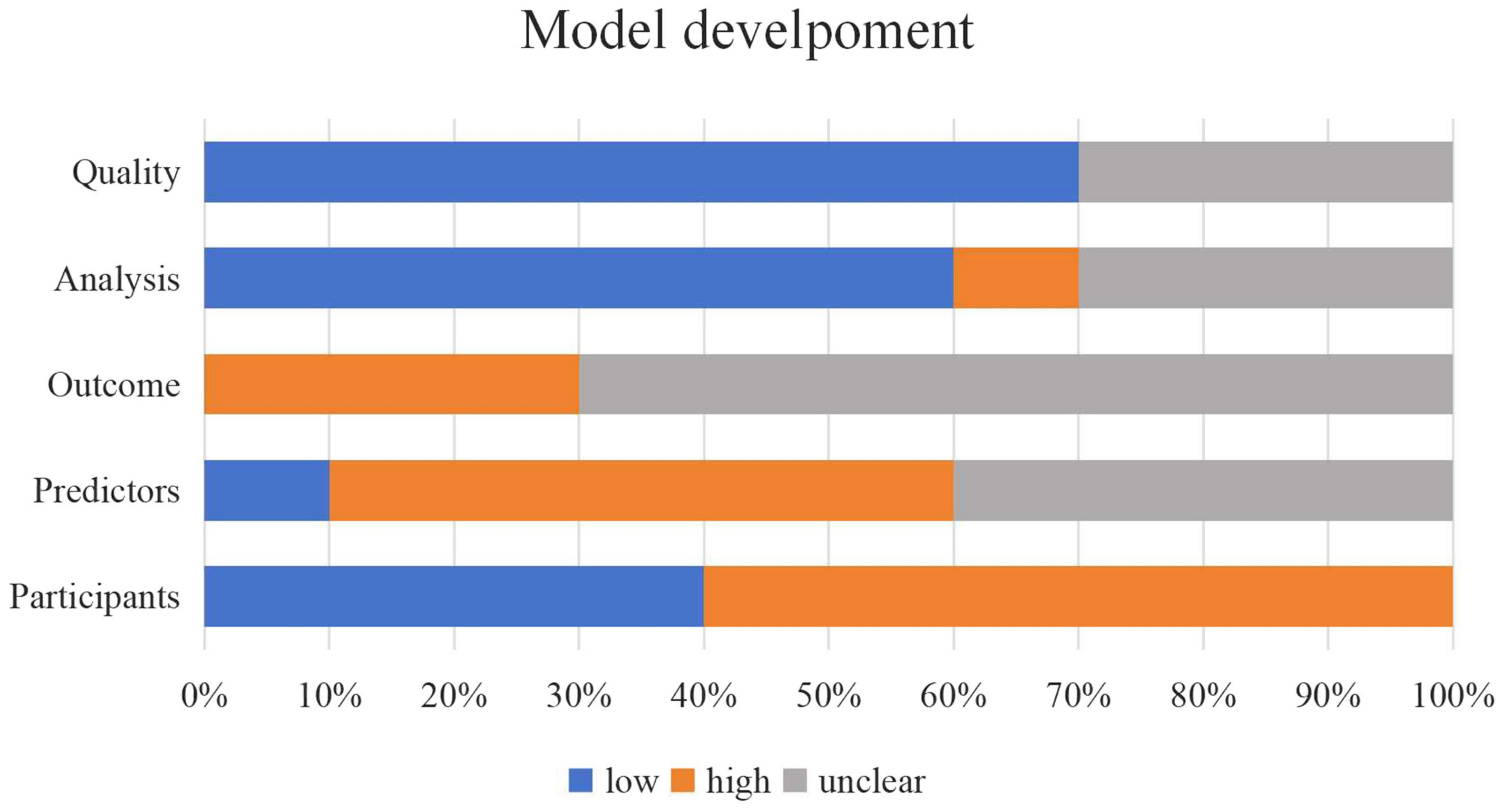
Bar chart of PROBAST+AI bias risk assessment results included in the study.

Two personnel used the PROBAST+AI assessment tool to evaluate the quality and applicability of the model development process and the risk of bias and applicability of the model performance assessment. After careful evaluation, in the model development and assessment section, seven studies had low-quality issues and high risk of bias concerns (31, 33, 34, 36–39) and the quality of the remaining three studies was rated as unclear (30, 32, 34). The results of applicability in the model development and assessment sections were the same, with low applicability of models in four studies (31, 33–35), high applicability of models in five studies (30, 32, 37–39), and unclear applicability of models in one study (36). The final results of bias and applicability of the included models are shown in Table 4. Bar charts showing the PROBAST+AI quality and risk of bias assessment results included in the study are presented in Figures 2, 3.

**Table 4:** Assess the overall concerns regarding quality, risk of bias and applicability of the prediction model.

Study	Model development		Model evaluation	
	Quality	Application	Risk of bias	Application
Mengjiao Yang 2024 (37)	+	−	+	−
Qinqin Liu 2023 (34)	?	+	?	+
Shaoyi Fan 2023 (31)	+	+	+	+
Yafei Wu 2022 (36)	+	?	+	?
Heeeun Jung 2023 (33)	+	+	+	+
Yin Yuan 2022 (38)	+	−	+	−
Yongfei Dong 2025 (30)	?	−	?	−
Lin Qi 2025 (35)	+	+	+	+
Wei Zhang 2024 (39)	+	−	+	−
Li Huang 2024 (32)	?	−	?	−

−, Low bias risk/high quality/high applicability; +, High bias risk/low quality/low applicability; ?, Unclear bias risk/unclear quality/unclear applicability.

In the model development and assessment section, the reasons for being rated as low quality and high bias were mainly attributed to (1) retrospective cohort studies raising low-quality concerns and high risk of bias among participants and data sources; (2) not reporting whether the measurements of the predictors were affected by outcomes that had already occurred; (3) the number of events per variable (EVP) was less than 20; (4) categorization of the continuous predictors; (5) missing data were directly excluded or information on missing data was not reported; (6) there was no information on methods to address category imbalance; and (7) there may be concerns about data leakage. Detailed assessment information is provided in Supplementary Table 4.

### 3.9 Statistical analysis

**Figure 4:**
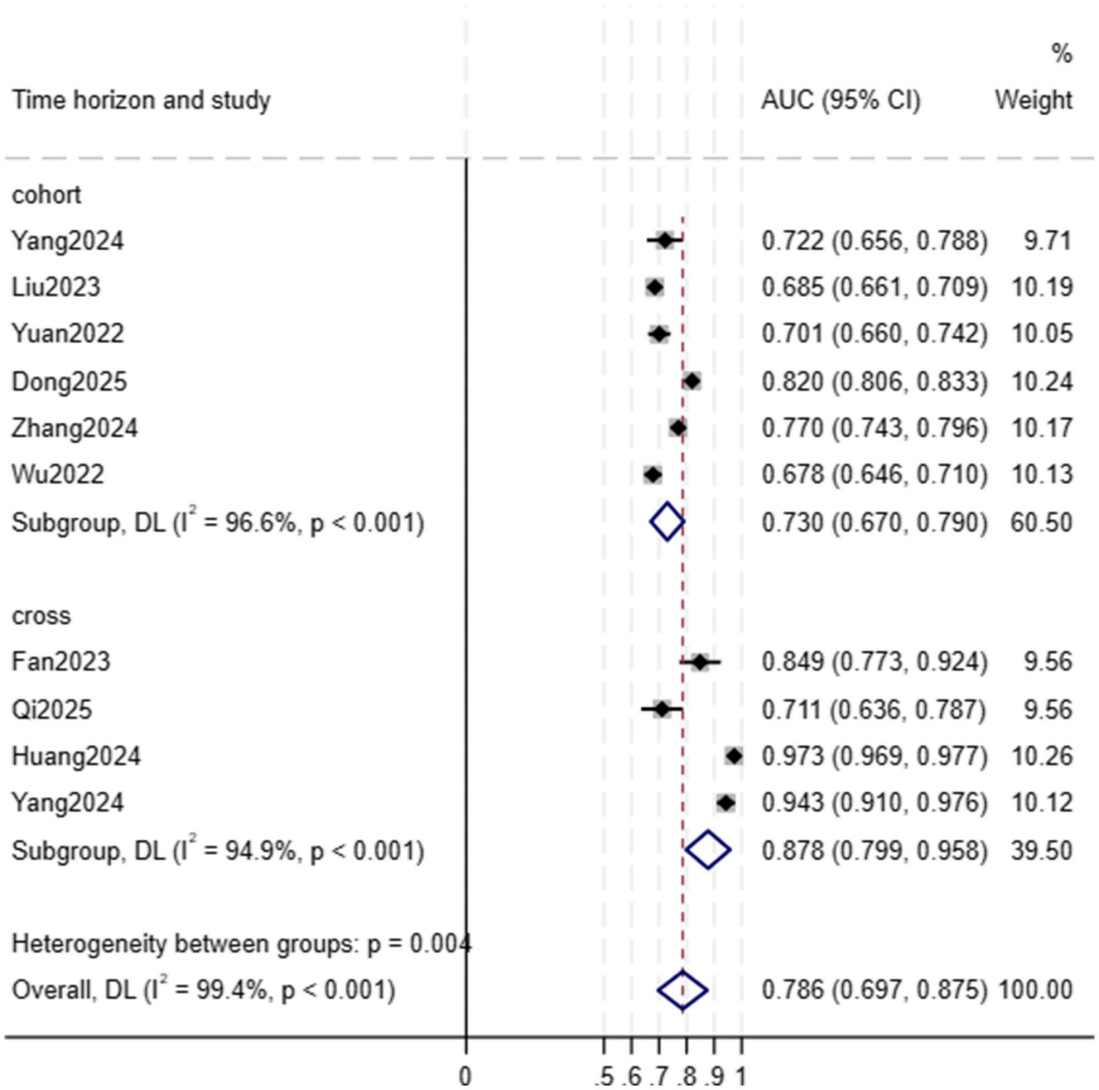
Random forest plot of area under the curve (AUC) values stratified by prediction window.

A total of 45 models were developed in the 10 included studies: 36 models were developed for internal validation, and nine models were developed for external validation. Since only three studies performed external validation and one study evaluated the performance of the model with the F1 score, the meta-analysis only included data from the internal validation set of 9 studies. Because most of the included studies used multiple ML models, we first summarized the AUC for each study, as shown in Supplementary Table 5. Given the variation in prediction time points across studies, we further conducted a stratified meta-analysis based on prediction windows (baseline prediction vs. longitudinal prediction). Results showed that the pooled AUC for baseline frailty prediction studies was 0.878 (95% CI 0.799, 0.958; *I*^2^ = 94.9%; heterogeneity *p* < 0.001), while the pooled AUC for longitudinal frailty prediction studies was 0.730 (0.670, 0.790; *I*^2^ = 96.6%; heterogeneity *p* < 0.001). The overall pooled AUC for all studies, regardless of prediction time point, was 0.786 (0.697, 0.875; *I*^2^ = 99.4%; heterogeneity *p* < 0.001). These findings indicate that frailty prediction models based on baseline data demonstrate superior discriminatory performance, with a significantly higher pooled AUC compared to longitudinal prediction studies. The results of the meta-analysis are shown in Figure 4.

Based on the different algorithms of model, we categorize them into traditional regression models (*n* = 4) as well as ML models (*n* = 28). Among them, the traditional regression models include LR, and the ML models include RF, SVM, XGB, etc. After summarizing the AUC of these models, we found that the combined AUC of the traditional regression model was 0.785 (95% CI 0.615, 0.955; *I*^2^ = 99.2%; heterogeneity *p* < 0.001), and the combined AUC of the ML model was 0.784 (95% CI 0.747, 0.822; *I*^2^ = 99.1%; heterogeneity *p* < 0.001). The results showed that the combined AUC values of the two models were similar. However, due to the smaller sample size of the traditional regression model, its CI was wider, suggesting higher uncertainty in the results. The results of the meta-analysis are shown in Supplementary Figure 1.

**Figure 5:**
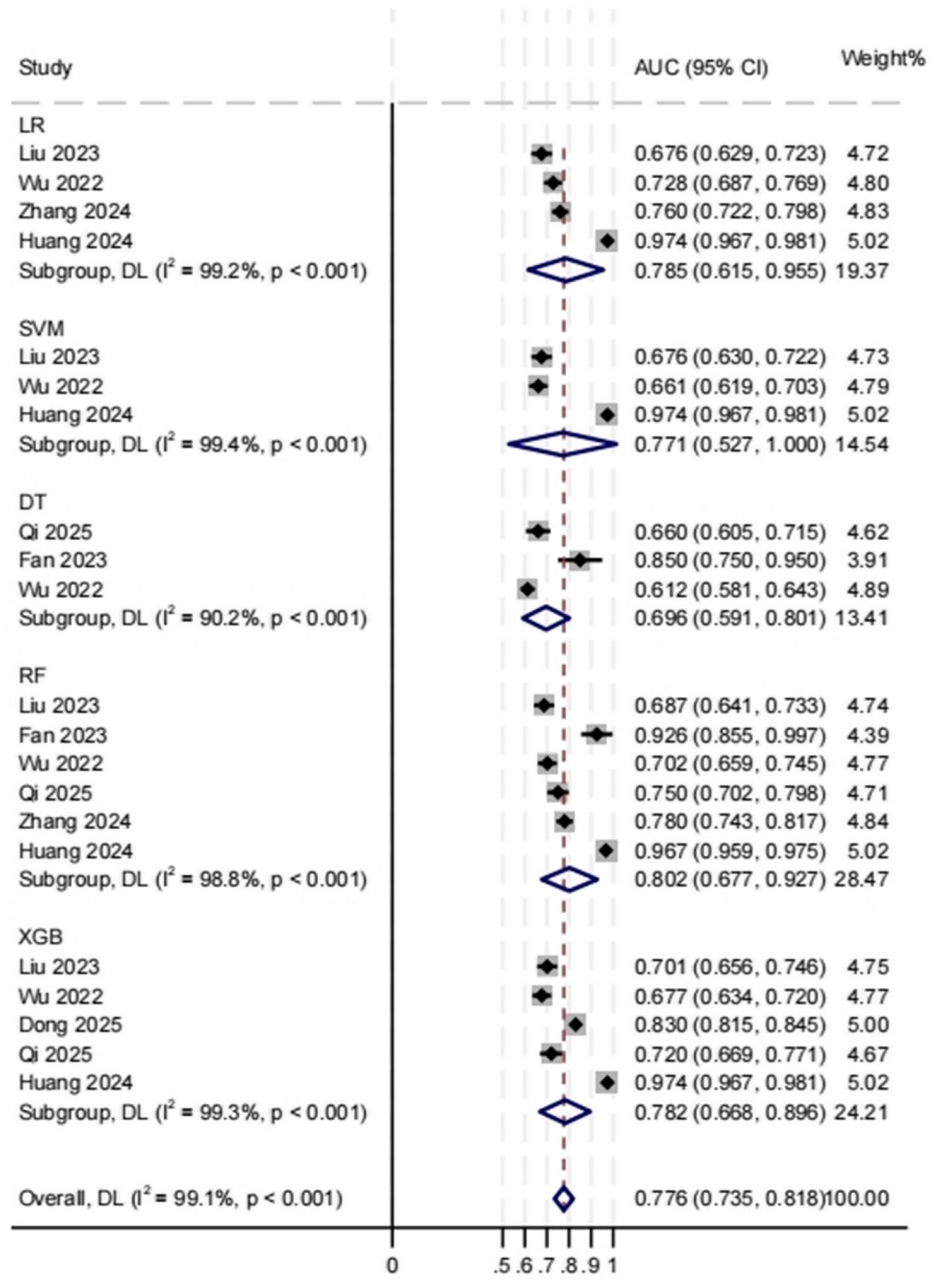
Random-effects forest plot of the area under the curve (AUC) values with 95% confidence intervals (CIs) for machine learning (ML) types.

To determine which ML model in the set of 9 internal validations had the better performance ability, we pooled and compared the AUCs of the models that were used three or more times. The results of the combined AUCs of the different ML models were as follows: 0.785 (95% CI 0.615, 0.955; *I*^2^ = 99.2%; heterogeneity *p* < 0.001); SVM, 0.711 (95% CI 0.527, 1; *I*^2^ = 99.4%; heterogeneity *p* < 0.001); DT, 0.696 (95% CI 0.591, 0.801; *I*^2^ = 90.2%; heterogeneity *p* < 0.001); RF, 0.802 (95% CI 0.677, 0.927; *I*^2^ = 98.8%; heterogeneity *p* < 0.001); XGB. 0.782 (95% CI 0.668, 0.896; *I*^2^ = 99.3%; heterogeneity *p* < 0.001). Among these models, RF had the best performance, while DT had a lower performance. However, these differences were not statistically significant. The results of the meta-analysis are shown in Figure 5.

### 3.10 Sensitivity analysis

This study included a total of 9 research projects. Sensitivity analysis indicated that the pooled results demonstrated good robustness. Forest plot results showed that the point estimates for all individual studies, after exclusion, clustered closely around the overall pooled effect size (between 0.72 and 0.74), with high overlap in confidence intervals. The results of the sensitivity analysis are shown in Supplementary Figure 2.

## 4 Discussion

This study reviewed machine-learning-based methods for constructing risk prediction models for the occurrence of frailty in community-dwelling older adults. The aim was to assess the quality of these studies and the performance of the machine learning predictive models to assist in the development of frailty screening tools and interventions for community-dwelling older adults.

### 4.1 Comparison of baseline prediction and longitudinal prediction

This study revealed through stratified meta-analysis that the performance of frailty prediction models based on baseline data (AUC = 0.878; 95% CI 0.799, 0.958) exceeded that of longitudinal prediction models (AUC = 0.730; 95% CI 0.670, 0.790). This performance difference stems not from inherent model superiority but from the fundamental distinction between the two predictive tasks.

In baseline prediction models, all predictors and frailty status are measured at the same point in time. While this feature helps the model identify individuals who are frail at baseline, it also leads to construct overlap between some predictors and the frailty phenotype itself, thereby introducing endogeneity issues (22). When predictors conceptually overlap with frailty or exhibit reverse causality due to simultaneous measurement, non-independence between predictors and frailty outcomes may occur, leading to overestimation of model performance (40). Consequently, baseline prediction models are better suited for screening and case identification, enabling the rapid detection of individuals who are currently in a frail state.

In contrast, the longitudinal prediction model seeks to forecast future events. During follow-up, subjects may experience competing risk events such as death, other illnesses, or lifestyle changes that influence frailty development. These events introduce random variability that is not accounted for by the model, objectively limiting its performance (41). Consequently, longitudinal predictive models perform below baseline models; yet, their clinical value lies primarily in their risk assessment and primary prevention capabilities. However, longitudinal prediction allows tracking and evaluation over time, yielding more accurate assessments of a model’s performance and effectiveness on a long-term timescale (32, 42). This enables the identification of currently healthy individuals at high risk of developing frailty in the future, facilitating early interventions to prevent or delay the onset of frailty.

In summary, the two models address distinct clinical questions, and their performance differences stem not only from randomness in follow-up processes but also from underlying endogeneity mechanisms within the data structure. Therefore, a model’s value should not be measured solely by AUC scores but interpreted based on its specific design objectives and application scenarios. Future research may utilize variables from non-frailty performance components as predictors, or consider methods such as mediation analysis and joint modeling to generate inputs with greater causal significance. This approach could reduce bias arising from endogeneity, thereby enhancing the conceptual rigor and technical validity of predictive models.

### 4.2 Research on the application of machine learning for predicting frailty in community-dwelling older adults remains in its exploratory phase

Without distinguishing prediction time points, the pooled AUC across all studies was 0.786 (95% CI 0.697, 0.875). This suggests that machine learning shows promising performance in predicting frailty onset among community-dwelling older adults, although the pooled AUC exhibits substantial heterogeneity. PROBAST+AI results suggest that most current studies raise concerns about low quality and high risk of bias. These issues may lead to potential overestimation of model performance metrics in this meta-analysis, while reducing the robustness and generalizability of the findings. Therefore, future research requires further standardization and improvement in study populations, data sources, predictive factors, and data analysis methodologies.

#### 4.2.1 Study population and data sources

In the included studies, most data originated from single-center databases with relatively limited sample sources, which may restrict the applicability of the model to other populations. In predictive modeling, prospective cohort studies are considered to be more advantageous due to their ability to provide the best measure of predictors and outcomes. In addition, the use of registered datasets as a source for modeling is thought to reduce bias (22). In contrast, using existing data and retrospective studies to construct models is not only susceptible to confounding factors. However, it may also cause recall bias, information bias, and selection bias, thus affecting the application of models in practice (43). In subsequent studies, it is recommended to prioritize multicenter, prospective samples for model development and validation to assess model performance across different settings and enhance model generalizability. When data originates from databases, details of data collection and measurement procedures should be reported. Additionally, researchers should clearly define the intended use of predictive models and establish appropriate inclusion and exclusion criteria.

#### 4.2.2 Differences in frailty assessment tools

Consistent with previous studies, the literature reviewed in this study revealed that the prevalence of frailty among the older adults in the community varied significantly (44), ranging from 13.00 to 64.77%. This difference may be due to the use of different assessment tools. Currently, although a variety of frailty assessment tools have been developed both domestically and abroad, there is no unified standard (45). Previous studies have shown that the reported prevalence of frailty is generally low due to the relatively strict criteria of tools such as SOF, FRAIL scale, Fried phenotype, and EFS; Scales such as CFS, VES-13, TFI, GFI, and FI tend to report a higher prevalence of frailty (46, 47). In community settings, the FRAIL scale is considered the most effective screening tool for distinguishing between frail and non-frail individuals, particularly in larger populations (48, 49). In this paper, a total of five frailty assessment tools is involved, although they are widely used in clinical research; however, the results may be inconsistent due to differences in assessment content.

Additionally, the definition of the debilitating outcome is a key factor influencing prevalence. Studies have shown that binary classifications that distinguish only between “frailty” and “non-frailty” tend to produce a higher prevalence of frailty than a trichotomy that includes “pre-frailty” states (50). Of the included studies, only five used binary classification to define outcomes. Additionally, one study combined ‘pre-frailty’ with ‘frailty’ as a predictive outcome, both of which may have influenced the results. Therefore, when developing a risk prediction model for frailty in the future, researchers should adopt more accurate methods for assessing and classifying frailty.

#### 4.2.3 Data analysis

First, among the included studies, three were deemed to have insufficient sample size due to an EPV < 20. PROBAST+AI indicates that in predictive model development, an EPV < 20 may increase the risk of model overfitting, thereby leading to an overestimation of the predictive model’s performance. Relevant studies have shown that when using machine learning methods to develop risk prediction models, a larger sample size is needed so that a small amount of overfitting can be achieved to ensure the stability of the developed model. When developing and validating predictive models, researchers are advised to calculate the required sample size based on the prevalence of the overall event, the number of candidate predictors, the model’s anticipated overall performance, and the target maximum level of overfitting. This approach helps reduce model overfitting and enhances model robustness (51). Second, the continuum predictors in the four studies were inappropriately converted to categorical variables, which may lead to missing information as well as spurious exchange effects that weaken the discriminatory power and calibration of the models (52, 53). During the development of predictive models, unnecessary grouping of continuous variables should be avoided whenever possible. Continuous variables should be incorporated into the model in their original form or converted into ordinal variables with clearly defined classification criteria.

In terms of the treatment of missing data, seven studies reported methods for handling missing data, while two studies deleted missing data. But even a small amount of missing or deleted data may introduce selective bias, reduce the representativeness of the sample, and may affect the judgment of model validity (54). PROBAST+AI noted that multiple interpolation was considered the preferred method of dealing with missing data, in addition to pattern-mixing models, proxy splitting, and other alternative strategies to avoid the need for interpolation, which can prevent biased results by dealing with missing data directly during the construction and validation of the predictive model (55). In one study, miss Forest was employed to address missing data, and this method, although highly predictive, may yield severely biased regression coefficient estimates and downwardly biased confidence interval coverage (56). Future researchers developing frailty prediction models should employ reasonable methods to handle missing data based on professional expertise, clearly reporting the proportion of missing data and the processing strategy to enhance model performance.

This study reveals that data leakage represents a prevalent yet underappreciated methodological flaw in this field, undermining the reliability of model performance evaluation. It frequently leads to overestimation of generalization capabilities, resulting in outcomes that cannot be accurately replicated in real-world scenarios (57, 58). Applying SMOTE (Synthetic Minority Over-sampling Technique) to the entire dataset before splitting it into training and test sets will cause overlapping leakage, thereby distorting model evaluation metrics. Among the included studies, two explicitly reported applying SMOTE oversampling only within the training set, effectively avoiding this type of information leakage (58). On the other hand, preprocessing operations (such as missing value imputation or feature selection) performed on the entire dataset before splitting it into training and test sets can introduce preprocessing leakage (59). Specifically, while all six included studies reported handling missing data, they failed to clarify the sequence of randomization versus data processing or disclose the imputation strategy. This methodological ambiguity elevates the risk of preprocessing leakage in their analyses. Therefore, future studies should prioritize data workflow standardization by adhering to the “split first, preprocess later” principle. Detailed data processing steps must be clearly documented in publications, particularly the methods for isolating training and test sets. This approach effectively prevents data leakage, enhances model reproducibility, and improves generalization performance.

Calibration and discrimination are two key metrics for evaluating predictive model performance. In the included studies, most models employed AUC to assess model discrimination, yielding favorable performance results. However, even with good discrimination, estimated risks may remain unreliable (60). Model calibration, which assesses the consistency between predicted risks and observed outcomes (61), is crucial for the validity and safety of clinical prediction models (62). Yet in practice, model calibration is often overlooked. Similarly, among the studies we included, only five reported model calibration. Furthermore, research suggests using decision curve analysis to evaluate model performance (e.g., net benefit). Decision curves comprehensively consider sensitivity, specificity, and the risk–benefit balance at different thresholds, providing crucial evidence for assessing a model’s value in real-world clinical settings (63). However, even when a model demonstrates strong discriminatory performance, its net benefit may diminish due to poor calibration (61). Therefore, future research should comprehensively evaluate model performance using multiple metrics—including discriminatory ability, calibration, and net benefit—to enhance reliability and practicality in real-world applications.

In predictive modeling research, external validation is often considered a critical step for predictive models to be applied in real-world practice. However, external validation using completely independent datasets is often overlooked (64). Of the 10 studies we included, only 3 performed external validation, while the rest performed only internal validation. The lack of external validation may have implications for model generalizability and robustness assessment (65). External validation, unlike model development, not only evaluates the model performance on a new dataset but also tests its adaptability to different environments or different datasets, thus demonstrating the generalizability and portability of the model (20). In addition, external validation can reveal whether the models in the training set are overfitted or not, as well as selectivity bias in the internal validation set. Models performing well in internal validation but with significant performance degradation in external validation (‘performance decay’) is a common phenomenon in predictive modeling studies (66). Therefore, the direct application of unvalidated models to clinical practice or public health screening is hazardous. Future research can evaluate model performance across diverse geographic regions, social contexts, and healthcare systems through time-geographic validation and multi-center validation at the institutional level, thereby enhancing the model’s fairness, generalizability, and external applicability. Furthermore, external validation of existing predictive models can reduce research waste and help bridge the gap between model development and implementation (67).

### 4.3 Interpretability of predictive models and key predictors

The studies we included employed various methods to enhance interpretability. Feature importance, the most common approach, enables researchers to identify key predictors influencing the model rapidly (68). However, it struggles to explain complex interactions between features, thereby limiting the depth of model interpretation and clinical applicability (69, 70). In contrast, Bayesian networks, as inherently interpretable models, reveal conditional dependencies between variables. They can identify potential causal relationships based on conditional dependencies and independence among variables (71). However, their construction accuracy relies on prior knowledge and data quality, which limits their practical application (72). Furthermore, SHapley Additive exPlanations (SHAP) stands as one of the most widely adopted explainable AI methods today. Its strength lies in simultaneously providing global feature importance and locally revealing key features and logical relationships underlying prediction outcomes. By quantifying the contribution of each feature to specific predictions, SHAP enhances model transparency and clinical interpretability (73, 74). Future research may develop multi-level interpretability frameworks that simultaneously report global feature importance and local prediction explanations. This approach could enhance model transparency and clinical applicability while maintaining predictive performance.

Based on the above analysis, we synthesized the interpretable findings from the included studies. The final results encompassed seven distinct categories of predictors: demographic data, socioeconomic factors, physical function and fitness indicators, cognitive and mental health, chronic diseases and medical conditions, nutritional and metabolic status, and social support. The heterogeneity of these predictors reflects that frailty arises from the combined effects of multiple influencing factors. Notably, across different explanatory methodologies, age, cognitive function, and activities of daily living (ADL) ability were the most frequently identified predictors. In most studies, age was recognized as a key predictor of frailty. Cognitive decline was also identified as a critical predictor of frailty progression. Research indicates a bidirectional relationship between cognitive impairment and physical functional decline, with both factors jointly accelerating the onset and progression of frailty (75). Furthermore, the combination of cognitive impairment and frailty may increase the risk of adverse health outcomes in older adults. Additionally, ADL is closely associated with frailty development. Previous studies suggest that ADL decline not only represents a clinical manifestation of frailty but may also serve as a mediating factor in its progression (5).

### 4.4 Comparison of predictive performance between machine learning models and traditional regression models

Given that ML is more efficient in dealing with complex nonlinear relationships and can improve prediction accuracy, we compared the performance of the ML model with that of the traditional regression model. Our results showed that the combined AUC point estimates of the traditional regression model and the ML model were similar, 0.785 (95% CI 0.615, 0.955) and 0.784 (95% CI 0.747, 0.822), respectively. However, due to the lack of statistical efficacy of the traditional regression group, which contained only four models at the time of the analysis, it is not yet possible to conclude that there is no difference in the performance of the two types of methods. Such small subgroups cannot reliably support the “no difference” conclusion, and their wide confidence intervals may improve the truthfulness, ranging from significantly worse to better than the ML models (76). Thus, the current results do not negate the theoretical advantages of ML in dealing with complex relationships. However, it suggests that logistic regression models are still competitive in specific scenarios due to their simplicity, stability, and ease of interpretation (77). Future research should fairly compare the advantages and disadvantages of different algorithms on the same high-quality dataset while ensuring methodological rigor.

## 5 Future outlook

This study provides a systematic evaluation of machine learning models for predicting frailty in community-dwelling older adults, offering insights for the development of healthcare policy and clinical practice. In clinical settings, the key predictors identified through interpretability analysis provide evidence-based support for developing concise and efficient frailty screening tools, enabling primary care providers to identify high-risk individuals rapidly. Furthermore, machine learning’s capability to handle complex nonlinear relationships among variables holds promise for future integration with primary healthcare management systems or electronic health records. This integration could enable the automated assessment of frailty risk and the development of early warning systems, thereby supporting the creation of personalized intervention plans tailored to individual needs.

However, the field currently faces challenges, including limited sample sizes, inadequate data processing methods, and insufficient external validation, which constrain model performance and clinical translation potential. To propel further research development, we recommend strengthening future studies in the following areas: (1) In terms of study design, encourage the conduct of multicenter prospective cohort studies to enhance data representativeness and model robustness; (2) Regarding data processing, it is recommended to adopt the TRIPOD+AI standard to enhance the transparency of methodology reporting, thereby ensuring the scientific rigor of model construction and the credibility of results.; (3) For predictors, clearly report their definitions, evaluation methods, and inclusion criteria to avoid spurious associations caused by ambiguous or non-standard variables; (4) For model validation, place greater emphasis on external validation by testing model performance on independent cohorts to enhance generalization and clinical applicability; (5) Regarding clinical translation, future studies should evaluate models’ practical clinical impact through prospective studies and randomized controlled trials, advancing their algorithms toward truly usable clinical tools.

## 6 Strengths and limitations

In this review, we conducted a systematic review and meta-analysis of machine learning methods to develop a predictive model of frailty risk in older adults in the community. In order to provide guidance for the development and update of subsequent models, and to help the application of such models in clinical practice. However, there were the following limitations in this study: (1) insufficient external validation: most of the included studies lacked external validation, so we were unable to perform meta-analysis of the pooled AUC of the model on the external validation set, which may affect the applicability and generalization of the model; (2) heterogeneity of assessment methods: the included studies had differences in the selection and measurement of frailty assessment methods and their predictors, which may introduce potential bias in the results of meta-analysis; (3) Heterogeneity between studies: Meta-analyses showed that there was in-house heterogeneity between studies, which may include differences between participants and study designs, predictor sets, and data analysis methods.

## 7 Conclusion

This study included a total of 10 original studies, with meta-analyses conducted on the internal validation sets of 9 studies. Among these, the pooled AUC for baseline frailty prediction studies was 0.878 (95% CI 0.799, 0.958), while the pooled AUC for longitudinal frailty prediction studies was 0.730 (95% CI 0.670, 0.790). When all studies were pooled without distinguishing prediction time points, the overall pooled AUC was 0.786 (95% CI 0.697, 0.875). This indicates that current machine learning-based predictive models for frailty onset in community-dwelling older adults demonstrate satisfactory performance. However, the 10 included studies were rated as low quality in model construction, high risk of bias in model validation, and lack of external validation for most of the models due to a lack of sufficient rigor in the model development process. Future studies should validate them through large-scale, multicenter prospective cohort studies with external validation to enhance the applicability of the models. Also, future studies should strictly adhere to the TRIPOD+AI reporting specifications and PROBAST+AI design specifications. This includes but is not limited to, detailed reporting of data sources, predictor definitions and measurements, missing data handling processes, sample size justification, category imbalance handling methods, strict data leakage prevention measures (explicit preprocessing after training–testing split), model tuning details, multiple performance metrics (discriminatory, calibration, and clinical utility), details of internal validation methodology, and conducting and reporting on independent external validation. Complete and transparent reporting and rigorous methodological design are cornerstones for improving study quality, reducing the risk of bias, and enhancing confidence and reproducibility of results.

## Data Availability

The original contributions presented in the study are included in the article/Supplementary material, further inquiries can be directed to the corresponding author.
