# Supplementary material for: Prediction of frailty in community older adults based on machine learning: a systematic review and meta-analysis
Source: Front Public Health. 2026 Jan 12;13:1667792. doi: 10.3389/fpubh.2025.1667792 (PMC12832267; doi:10.3389/fpubh.2025.1667792)
Supplement: Supplementary file 1 [file Table_1.docx]

Supplementary Material

# Supplementary Figures and Tables

## Supplementary Figures


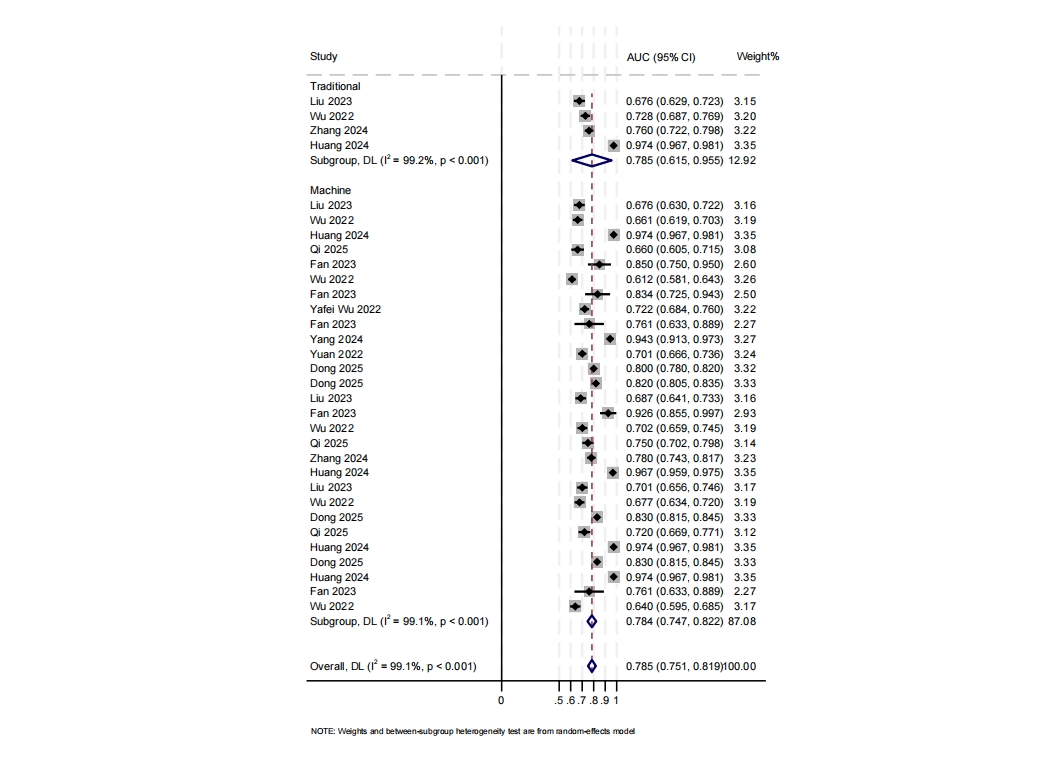


**Supplementary Figure 1** Random effects forest plot of AUC in ML model type

**Supplementary Figure 2** The sensitivity analysis

# 2.1Supplementary Tables

**Table S1 Search strategy and search terms**

| Database | Search terms |
| --- | --- |
| PubMed | #1((((((frailty[MeSH Terms]) OR (asthenia[MeSH Terms])) OR (“frail elderly”[Title/Abstract])) OR (frailty*[Title/Abstract])) OR (frail[Title/Abstract])) OR (“frailty syndrome”[Title/Abstract])) OR (debility*[Title/Abstract])  #2((community[Title/Abstract]) OR ("community dwelling"[Title/Abstract])) OR ("community dweller"[Title/Abstract])  #3(((aged[MeSH Terms]) OR #3(aged[Title/Abstract])) OR (older[Title/Abstract])) OR (elder*[Title/Abstract])  #4((((((((("prediction model") OR ("Predictive Learning Models")) OR (model)) OR ("risk factor")) OR ("predictor")) OR ("risk assessment")) OR ("risk prediction")) OR ("risk prediction model")) OR ("risk score")) OR (" prediction model") OR (“Machine Learning”)) OR (“Artificial Intelligence”))  #5 #1 AND #2 AND #3 AND #4 |
| Web of science | #1TS=(frailty* OR frail OR asthenia OR frail elderly OR frailty syndrome OR debility*)and Preprint Citation Index (Exclude – Database)  #2TS=(community OR "community dwelling" OR "community dweller") and Preprint Citation Index (Exclude – Database)  #3 TS=(aged OR older OR elder) and Preprint Citation Index (Exclude – Database  #4 TS=("prediction model" OR "Predictive Learning Models" OR model OR "risk factor" OR "predictor" OR "risk assessment" OR "risk prediction" OR "risk prediction model" OR "risk score" OR "risk prediction model" OR “Artificial Intelligence” OR “Machine Learning”) and Preprint Citation Index (Exclude – Database)  #5 #1 AND #2 AND #3 AND #4 and Preprint Citation Index (Exclude – Database) |
| Embase | #1 (“frail elderly” OR “frail” OR “frailty” OR “asthenia” OR “frailty syndrome” OR “debility*”) ab,ti,kw  #2 (community OR "community dwelling" OR "community dweller") ab,ti,kw  #3 (aged OR older OR elder) ab,ti,kw  #4 ("prediction model" OR "Predictive Learning Models" OR model OR "risk factor" OR "predictor" OR "risk assessment" OR "risk prediction" OR "risk prediction model" OR "risk score" OR "risk prediction model" OR “Machine Learning” OR “Artificial Intelligence”)All fields  #5 #1 AND #2 AND #3 AND #4 |
| Cocrhane | #1 (“frail” OR “frailty”OR “asthenia” OR “frailty syndrome” OR “debility*”) ab,ti,kw  #2(“community” OR "community dwelling" OR "community dweller") ab,ti,kw  #3(aged OR older OR elder) ab,ti,kw  #4 ("prediction model" OR "Predictive Learning Models" OR model OR "risk factor" OR "predictor" OR "risk assessment" OR "risk prediction" OR "risk prediction model" OR "risk score" OR "risk prediction model" OR “Artificial Intelligence” OR “Machine Learning”)All Text  #5 #1 AND #2 AND #3 AND #4 |
| Scopus | ( TITLE-ABS-KEY ( aged OR elderly OR older ) ) AND ( TITLE-ABS-KEY ( “frail” OR “frailty”OR “asthenia” OR “frailty syndrome” OR “debility*” ) ) AND ( TITLE-ABS-KEY ( community OR community-dwelling OR "community dweller" ) ) AND ( TITLE-ABS-KEY ( "prediction model" OR "Predictive Learning Models" OR model OR "risk factor" OR "predictor" OR "risk assessment" OR "risk prediction" OR "risk prediction model" OR "risk score" OR "risk prediction model" OR “Artificial Intelligence” OR “Machine Learning” ) ) |
| CINAHL | #1 (“frail” OR “frailty”OR “asthenia” OR “frailty syndrome” OR “debility*”) ab  #2 (“community” OR "community dwelling" OR "community dweller") ab  #3 (aged OR older OR elder) ab  #4 ("prediction model" OR "Predictive Learning Models" OR model OR "risk factor" OR "predictor" OR "risk assessment" OR "risk prediction" OR "risk prediction model" OR "risk score" OR "risk prediction model" OR “Machine Learning” OR “Artificial Intelligence”)All Text  #5 #1 AND #2 AND #3 AND #4 |
| CKNI | #1(篇关摘：老人)OR (篇关摘：老年人)  Translate:(TKA：older) OR (TKA:elder)  #2(篇关摘：衰弱) OR (篇关摘：虚弱) OR (篇关摘：脆弱) OR (篇关摘：老年人衰弱) OR (篇关摘：衰弱综合征)  Translate:(TKA:frail) OR (TKA:weakness) OR (TKA  : “frailty syndrome”) OR (TKA:“elderly frailty”)  #3(篇关摘：社区） OR (篇关摘：社区居住) OR  Translate:(TKA=community) OR (TKA:"community dwelling")  #4:(主题：预测模型 OR 模型 OR 风险因素 OR 预测因子 OR 风险评估 OR 风险预测 OR 风险预测模型 OR 风险得分 OR 人工智能 OR 机器学习)  Translate:(SU=prediction model) OR (SU:model) OR (SU:risk factor) OR (SU:predictor) OR (SU:risk assessment) OR (SU:risk prediction) OR (SU:risk prediction model) OR (SU:risk score) OR (SU:risk prediction model) OR (SU:Artificial Intelligence) OR (SU:Machine Learning)  #1 AND #2 AND #3 AND #4 |
| VIP | #1 M=(老人 OR 老年人)  Translate：M=(old man OR senium)  #2 M=(衰弱 OR 虚弱 OR 脆弱 OR 衰弱综合征 OR 老年人衰弱)  Translate:M=(frailty OR weakness OR frailty syndrome OR Vulnerability OR frailty syndrome OR elderly frailty)  #3 M=(社区 OR 社区居住）  M=(community OR "community dwelling")  #4 M=(预测模型 OR 模型 OR 风险因素 OR 预测因子 OR 风险评估 OR 风险预测 OR 风险预测模型 OR 风险得分 OR 机器学习 OR 人工智能)  M=("prediction model" OR model OR "risk factor" OR "predictor" OR "risk assessment" OR "risk prediction" OR "risk prediction model" OR "risk score" OR Artificial Intelligence OR Machine Learning))  #5 #1 AND #2 AND #3 AND #4 |
| Wanfang Database | #1 (主题：老人) OR (主题：老年人)  Translate:(Subject: old man) OR (Subject: senium)  #2 (主题：衰弱) OR (主题：虚弱) OR (主题：脆弱) OR (主题：衰弱综合征) OR (主题：老年人衰弱)  Translate:(Subject: Frailty) OR (Subject: Weakness) OR (Subject: Vulnerability) OR (Subject: Frailty Syndrome) OR (Subject: Frailty in the Elderly)  #3 (主题：社区) OR (主题：社区居住)  Translate:(Subject: Community) OR (Subject: Community dwelling)  #4(主题：预测模型) OR (主题：模型) OR (主题：风险因素) OR (主题;预测因子) OR (主题：风险评估) OR (主题：风险预测) OR (主题：风险预测模型) OR (主题：风险得分) OR (主题：机器学习) OR (主题：人工智能)  Translate：(Subject: Predictive Model) OR (Subject: Model) OR (Subject: Risk Factors) OR (Subject; Predictor OR (Topic: Risk Assessment) OR (Topic: Risk Prediction) OR (Topic: Risk Prediction Model) OR (Topic: Risk Score) OR (Topic: Machine Learning) OR (Topic: Artificial Intelligence)  #5 #1 AND #2 AND #3 and #4 |
| SinoMed | #1"老人"[常用字段:智能] OR "老年人"[常用字段:智能]  Translate:"Elderly Person "[Common field: Intelligent] OR" Senior Citizen "[Common field: Intelligent]  #2“衰弱”[常用字段:智能] OR “虚弱”[常用字段:智能] OR “脆弱”[常用字段:智能] OR “衰弱综合征”[常用字段:智能] OR “老年人衰弱”[常用字段:智能]  Translate:"Frailty" [Common Fields: Intelligent] OR "Weakness" [Common Fields: Intelligent] OR "Vulnerability" [Common Fields: Intelligent] OR "Frailty Syndrome" [Common Fields: Intelligent] OR "Frailty in the Elderly" [Common Fields: Intelligent]  #3“社区”[常用字段:智能] OR “社区居住”[常用字段:智能]  Translate:"Community" [Common field: Intelligent] OR "Community dwelling" [Common field: Intelligent]  #4“预测模型”[常用字段:智能] OR “模型”[常用字段:智能] OR “风险因素”[常用字段:智能] OR “预测因子”[常用字段:智能] OR “风险评估”[常用字段:智能] OR “风险预测”[常用字段:智能] OR “风险预测模型”[常用字段:智能] OR “风险得分”[常用字段:智能] OR “机器学习”[常用字段:智能] OR “人工智能”[常用字段:智能]  Translate:"Predictive Model" [Common Fields: Intelligent] OR "Model" [Common Fields: Intelligent] OR "Risk Factor" [Common Fields: Intelligent] OR "Predictor" [Common Fields: Intelligent] OR "Risk Assessment" [Common Fields: Intelligent] OR "Risk Prediction" [Common Fields: Intelligent] OR "Risk Prediction Model" [Common Field: Intelligent] OR "Risk Score" [Common Field: Intelligent] OR "Machine Learning" [Common Field: Intelligent] OR "Artificial Intelligence" [Common Field: Intelligent]  #5#1 AND #2 AND #3 AND #4 |

**Table S2** Detail of author、years and publisher

| **Author (year)** | **Years** | **Publisher** |
| --- | --- | --- |
| Mengjiao Yang | 2024 | BMC Public Health |
| Qinqin Liu | 2023 | International Journal of Medical Informatics |
| Shaoyi Fan | 2023 | Frontiers in Public Health |
| Yafei Wu | 2022 | BMC Geriatrics |
| Heeeun Jung | 2023 | International Conference of the IEEE Engineering in Medicine & Biology Society |
| Yin Yuan | 2022 | BMC Geriatrics |
| Yongfei Dong | 2025 | Public Health |
| Lin Qi | 2025 | Frontiers in Public Health |
| Wei Zhang | 2024 | Journal of Advanced Nursing |
| Li Huang | 2024 | Scientific Reports |

**Table S3** Interpretability analysis methods across studies and the top five key predictors identified

| Author (year) | Interpretability analysis method | The top five key predictors |
| --- | --- | --- |
| Mengjiao Yang  2024 | Bayesian Network | age、function status、social relationship、dement、polypharm |
| Qinqin Liu  2023 | Feature importance | RF：waist circumstance、age、cognitive function、self-rate health、material wealth  XGBoost：age、waist circumstance、cognitive function、medical insurance、self-rate health |
| Shaoyi Fan  2023 | SHAP | Walking speed,、average step size、age、total step walking distance,、MMSE |
| Yafei Wu  2022 | SHAP | IADL、marital status、ADL、MMSE、hypertension |
| Heeeun Jung  2023 | Feature importance | timed up and go、short form performance battery、EuroQol-5 Dimension、depressive symptom、age |
| Yin Yuan  2022 | Bayesian Network | ADL/IADL、MNA-SF score、HDL-C、DBP  hospitalization. |
| Yongfei Dong  2025 | Nomogram | age、MMSE、BMI、sex、nationality |
| Lin Qi  2025 | Feature importance | RF：sleep Duration、type of medication  age、visit frequency、BMI  XGBoost：type of medication、number of chronic diseases、living arrangement、pension insurance、BMI |
| Wei Zhang | Feature importance  Nomogram | Feature importance：age、ADL、MMSE、income、sleep duration  Nomogram：age、residence、marriage、self-report health、MMSE |
| Li Huang  2024 | Feature importance | Able to go shopping by yourself?  Able to walk one kilometer?  Able to carry 5kg weight?  Able to make food by yourself?  Able to crouch and stand for three times? |

**Note:**ADL,activities of daily living;MMSE, Mini-Mental the State Examination;IADL,instrumental activities of daily living;BMI,body mass index;HDL-C,high-density lipoprotein cholesterol;DBP,diastolic blood pressure

**Table S4** Assess quality and applicability or risk of bias and applicability

**Table S4.1** PROBAST+AI model development.

| Author  (year) | Participants and data sources | | | | Predictors | | | | | Outcome | | | | | Analysis | | | | | | Overall |
| --- | --- | --- | --- | --- | --- | --- | --- | --- | --- | --- | --- | --- | --- | --- | --- | --- | --- | --- | --- | --- | --- |
|  | 1.1 | 1.2 | 1.3 |  | 2.1 | 2.2 | 2.3 | 2.4 |  | 3.1 | 3.2 | 3.3 | 3.4 |  | 4.1 | 4.2 | 4.3 | 4.4 | 4.5 |  |  |
| Mengjiao Yang  2024 | PN | Y | Y | - | Y | Y | Y | Y | - | Y | Y | NI | Y | ？ | N | N | PN | NI | Y | + | + |
| Qinqin Liu  2023 | Y | Y | Y | - | Y | Y | Y | Y | ? | N | Y | NI | Y | - | Y | Y | PN | NI | Y | ? | ? |
| Shaoyi Fan  2023 | Y | Y | N | + | Y | Y | Y | Y | + | Y | Y | NI | Y | - | N | Y | PN | N | Y | + | + |
| Yafei Wu  2022 | Y | N | Y | + | NI | Y | Y | Y | ? | Y | Y | NI | Y | ? | Y | Y | PN | Y | Y | - | + |
| Heeeun Jung  2023 | PN | Y | Y | - | NI | Y | Y | Y | ? | Y | Y | NI | Y | ？ | N | Y | N | Y | Y | + | + |
| Yin Yuan  2022 | Y | Y | N | + | Y | Y | Y | Y | - | Y | Y | NI | Y | ？ | Y | N | NI | NI | Y | + | + |
| Yongfei Dong  2025 | Y | Y | Y | - | Y | Y | Y | Y | - | Y | Y | NI | Y | ？ | Y | Y | NI | NI | NI | ? | ? |
| Lin Qi  2025 | PN | Y | Y | + | Y | Y | Y | Y | - | Y | Y | NI | Y | ？ | Y | N | N | NI | Y | + | + |
| Wei Zhang  2024 | Y | Y | Y | - | NI | Y | Y | Y | ？ | Y | Y | Y | Y | - | Y | Y | PN | NI | Y | + | + |
| Li Huang  2024 | Y | Y | Y | - | Y | Y | Y | Y | - | Y | Y | NI | Y | ? | Y | N | NI | NI | Y | ? | ? |

**Note:** -, High quality; +, Low quality;?, Not clear quality

**Table S4.2** PROBAST+ AI model evaluation

| Author  (year) | Participants and data sources | | | | Predictors | | | | | Outcome | | | | | Analysis(A Surface performance; I internal verification;E external verification） | | | | | | | | Overall |
| --- | --- | --- | --- | --- | --- | --- | --- | --- | --- | --- | --- | --- | --- | --- | --- | --- | --- | --- | --- | --- | --- | --- | --- |
|  | 1.1 | 1.2 | 1.3 |  | 2.1 | 2.2 | 2.3 | 2.4 |  | 3.1 | 3.2 | 3.3 | 3.4 |  | 4.1 | 4.2  (AIE) | 4.3  (AIE) | 4.4  (AIE) | 4.5  (I) | 4.6  (I) | 4.7  (AIE) |  |  |
| Mengjiao Yang  2024 | PN | Y | Y | - | Y | Y | Y | Y | - | Y | Y | NI | Y | ？ | Y | N/N | PN/PN | N/N | Y | Y | NI/Y | + | + |
| Qinqin Liu  2023 | Y | Y | Y | - | Y | Y | Y | Y | - | N | Y | NI | Y | ? | Y | Y/Y/Y | PN/PN/PN | NI/NI/NI | Y/Y/Y | Y/Y/Y | Y/Y/Y | ? | ? |
| Shaoyi Fan  2023 | Y | Y | N | + | Y | Y | Y | Y | - | Y | Y | NI | Y | ？ | Y | N/N | PN/PN | N/N | Y | NI | NI/Y | + | + |
| Yafei Wu  2022 | Y | N | Y | + | NI | Y | Y | Y | ？ | Y | Y | NI | Y | ？ | Y | Y/Y/Y | PN/PN/PN | Y/Y/Y | Y/Y/Y | Y/Y/Y | Y/Y/Y | + | + |
| Heeeun Jung  2023 | PN | Y | Y | - | NI | Y | Y | Y | ？ | Y | Y | NI | Y | ？ | Y | N/N | N/N | Y/N | Y | Y | NI/Y | + | + |
| Yin Yuan  2022 | Y | Y | N | + | Y | Y | Y | Y | - | Y | Y | NI | Y | ？ | Y | Y/Y | NI/NI | NI/NI | N | Y | NI/Y | + | + |
| Yongfei Dong  2025 | Y | Y | Y | - | Y | Y | Y | Y | - | Y | Y | NI | Y | ？ | Y | Y/Y/Y | NI/NI/NI | NI/NI/NI | Y | Y | Y/Y/Y | ? | ? |
| Lin Qi  2025 | PN | Y | Y | + | Y | Y | Y | Y | - | Y | Y | NI | Y | ？ | Y | Y/Y | N/N | NI/NI | Y | Y | Y | + | + |
| Wei Zhang  2024 | Y | Y | Y | - | NI | Y | Y | Y | ？ | Y | Y | Y | Y | - | Y | Y/Y | PN/PN | N/N | Y | Y | Y/Y | + | + |
| Li Huang  2024 | Y | Y | Y | - | Y | Y | Y | Y | - | Y | Y | NI | Y | ？ | Y | Y/Y | NI/NI | NI/NI | Y | Y | Y/Y | ? | ? |

**Note:** -, Low risk of bias; +, High risk of bias;?,Unclear risk of bias

**Table S4.3** Applicability Assessment

| Author  (year) | Model development | | | | Model evaluation | | | |
| --- | --- | --- | --- | --- | --- | --- | --- | --- |
|  | Participants and data sources | Predictors | Outcome | Overall | Participants and data sources | Predictors | Outcome | Overall |
| Mengjiao Yang  2024 | - | - | - | - | - | - | - | _ |
| Qinqin Liu  2023 | - | - | + | + | - | - | + | + |
| Shaoyi Fan  2023 | + | - | - | + | + | - | + | + |
| Yafei Wu  2022 | - | ? | - | ? | - | ? | - | ? |
| Heeeun Jung  2023 | - | + | - | + | - | + | - | + |
| Yin Yuan  2022 | - | - | - | - | - | - | - | _ |
| Yongfei Dong  2025 | - | - | - | - | - | - | - | _ |
| Lin Qi  2025 | + | - | - | + | - | - | - | + |
| Wei Zhang  2024 | - | - | - | - | - | - | - | - |
| Li Huang  2024 | - | - | - | - | - | - | - | - |

**Note:** -, High applicability; +, Low applicability;?, Unclear applicability

**Table S4** Pooled AUC of each included study

| **Study** | **Machine Learning** | **Pooled AUC 95% CI** |
| --- | --- | --- |
| Mengjiao Yang  2024 | Bayesian networks | Cross-section:0.943（0.913,0.974）  Longitudinal:0.722(0.656,0.788) |
| Qinqin Liu  2023 | LR，SVM，RF，XGB | 0.685（0.661,0.709） |
| Shaoyi Fan  2023 | RF，DT，NN，SGD，NB | 0.849（0.773,0.924） |
| Yafei Wu  2022 | NB，LR，DT，SVM，ANN，RF，XGB | 0.678（0.646，0.710） |
| Yin Yuan  2022 | Bayesian networks | 0.701（0.655,0.737） |
| Lin Qi  2025 | XGB，GBM，Cox model  ，COX Boost | 0.711（0.611,0.762） |
| Wei Zhang  2024 | RF，XGB，DT | 0.770（0.744,0.797） |
| Li Huang  2024 | RF，LR | 0.973（0.969,0.977） |
| Yongfei Dong  2025 | LR，RF，SVM，XGB，SHLNN | 0.820（0.807,0.834） |
